# Procoagulant, Tissue Factor-Bearing Microparticles in Bronchoalveolar Lavage of Interstitial Lung Disease Patients: An Observational Study

**DOI:** 10.1371/journal.pone.0095013

**Published:** 2014-04-28

**Authors:** Federica Novelli, Tommaso Neri, Laura Tavanti, Chiara Armani, Concettina Noce, Fabio Falaschi, Maria Laura Bartoli, Federica Martino, Antonio Palla, Alessandro Celi, Pierluigi Paggiaro

**Affiliations:** 1 Dipartimento di Patologia Chirurgica, Medica, Molecolare e dell’Area Critica, University of Pisa and Azienda Ospedaliero-Universitaria Pisana, Pisa, Italy; 2 U.O. Radiodiagnostica 2 SSN, Azienda Ospedaliero-Universitaria Pisana, Pisa, Italy; University of Illinois at Chicago, United States of America

## Abstract

Coagulation factor Xa appears involved in the pathogenesis of pulmonary fibrosis. Through its interaction with protease activated receptor-1, this protease signals myofibroblast differentiation in lung fibroblasts. Although fibrogenic stimuli induce factor X synthesis by alveolar cells, the mechanisms of local posttranslational factor X activation are not fully understood. Cell-derived microparticles are submicron vesicles involved in different physiological processes, including blood coagulation; they potentially activate factor X due to the exposure on their outer membrane of both phosphatidylserine and tissue factor. We postulated a role for procoagulant microparticles in the pathogenesis of interstitial lung diseases. Nineteen patients with interstitial lung diseases and 11 controls were studied. All subjects underwent bronchoalveolar lavage; interstitial lung disease patients also underwent pulmonary function tests and high resolution CT scan. Microparticles were enumerated in the bronchoalveolar lavage fluid with a solid-phase assay based on thrombin generation. Microparticles were also tested for tissue factor activity. In vitro shedding of microparticles upon incubation with H_2_O_2_ was assessed in the human alveolar cell line, A549 and in normal bronchial epithelial cells. Tissue factor synthesis was quantitated by real-time PCR. Total microparticle number and microparticle-associated tissue factor activity were increased in interstitial lung disease patients compared to controls (84±8 vs. 39±3 nM phosphatidylserine; 293±37 vs. 105±21 arbitrary units of tissue factor activity; mean±SEM; p<.05 for both comparisons). Microparticle-bound tissue factor activity was inversely correlated with lung function as assessed by both diffusion capacity and forced vital capacity (r^2^ = .27 and .31, respectively; p<.05 for both correlations). Exposure of lung epithelial cells to H_2_O_2_ caused an increase in microparticle-bound tissue factor without affecting tissue factor mRNA.

Procoagulant microparticles are increased in interstitial lung diseases and correlate with functional impairment. These structures might contribute to the activation of factor X and to the factor Xa-mediated fibrotic response in lung injury.

## Introduction

Interstitial lung diseases (ILDs) are a group of diseases resulting from damage to the lung parenchyma by a combination of inflammation and fibrosis [1]. A large number of ILDs are of unknown cause and are called idiopathic interstitial pneumonias (IIPs); the most frequent form of IIP, called idiopathic pulmonary fibrosis (IPF), is associated with a characteristic histopathologic and/or radiologic pattern called usual interstitial pneumonia (UIP) [2]. IPF is associated with a poor prognosis, with a median survival of 2 to 3 years from the time of diagnosis [2]. No therapeutic interventions have demonstrated an increase in survival time in randomized, controlled clinical trials.

Blood coagulation is a tightly regulated homeostatic reaction that ultimately leads to the generation of an insoluble matrix of fibrin, with the aim of preventing blood loss at sites of tissue injury. According to the classical model, blood coagulation is initiated through the so called extrinsic pathway by the contact of circulating factor (F) VIIa with membrane-associated tissue factor (TF) expressed by non vascular cells (hence extrinsic to blood) and exposed upon tissue injury; the FVIIa/TF complex activates FX to FXa, which in turn activates prothrombin to thrombin [3]. The observation that functional TF circulates in the bloodstream of normal individuals, however, has challenged this model [4]. One proposed alternate mechanism for the activation of the extrinsic pathway of blood coagulation is represented by circulating TF-bearing microparticles (MP) [5], [6]. MP are small (.05–1 µm) membrane vesicles shed by virtually all cells upon activation and/or apoptosis [7]. MP represent disseminated storage pools of bioactive effectors involved in a variety of physiologically relevant reactions [8] [9]–[11]. Relevant to pulmonary pathophysiology, Bastarache and coll. have demonstrated a role for procoagulant, alveolar epithelial cell-derived MP in the pathogenesis of the acute respiratory distress syndrome [12].

Increasing evidence suggests a role for the activation of the coagulation cascade in the pathogenesis of PF. In patients with IPF and PF secondary to systemic sclerosis, TF expression is upregulated on type II pneumocytes [13]. In the latter group of patients, thrombin is increased in bronchoalveolar lavage fluid (BALF) [14]. Further evidence for a role of the activation of blood coagulation stems from the observation that anticoagulants attenuate collagen deposition in a rat model of bleomycin-induced lung fibrosis [15]. From a clinical point of view, IPF patients are at increased risk for pulmonary thromboembolism [16]. Finally, a small, open label clinical trial has shown an increased 3-year survival in patients treated with anticoagulants (either warfarin or low molecular weight heparin) plus prednisone compared to the prednisone alone group [17], although a more recent, randomized trial with warfarin and no prednisone failed to confirm the result [18].

Besides their role in fibrin formation, FXa and thrombin also act as signaling molecules through their interaction with protease activated receptors (PAR) 1–4 [19]. Scotton and colleagues have investigated the role of FXa-PAR-1 interaction in PF. They showed an increase in FX expression in fibrotic lung tissue (both human and murine); they also demonstrated that FXa induces myofibroblast differentiation in cultured lung fibroblasts via PAR-1 mediated signaling. Agonists implicated in IPF pathogenesis, including the oxidant H_2_O_2_, increased FX expression at the mRNA level in A549 lung epithelial cells [20].

Since FX must be converted to FXa in order to participate in the coagulation cascade and to function as a signaling molecule via PAR-1, in an attempt to further clarify the mechanisms of FX activation in the context of PF, we investigated the potential implication of TF-bearing MP in the pathogenesis of the disease.

## Materials and Methods

### Ethics Statement

The study was approved by the institutional review board at the Azienda Ospedaliero-Universitaria Pisana, Pisa, Italy. All patients gave their written informed consent to the bronchoscopy as part of the Hospital standard procedure. However, no specific consent for data analysis was obtained since the data were analyzed anonymously; patient information was anonymized and de-identified prior to analysis. All clinical investigations have been conducted according to the principles expressed in the Declaration of Helsinki.

### Patients

The study included 19 patients with PF diagnosed between December 2010 and September 2012 and during the months of January and February 2014, and 11 control subjects, who underwent routine BAL for reasons different than PF. The definitive diagnosis of the latter were: pneumonia (n = 4), bronchiectasis (n = 3), hemoptysis without need for further characterization (n = 2), chronic left ventricular failure (n = 1), reactive mediastinal lymphadenopathy (n = 1). All patients gave their written consent to the procedure. The diagnosis of PF was performed according to the clinical manifestations and the presence of high resolution computed tomography (HRCT) signs of fibrosis: reticular opacity and/or traction bronchiectasis and/or honeycombing. One patient had asbestosis, one patient had PF in scleroderma, one patient had PF in polymyalgia, one patient had chronic hypersensitivity pneumonitis and 15 patients had IIP. Among the patients with IIP, 8 patients had an HRCT showing an UIP pattern diagnostic for IPF and 7 patients had an HRCT with signs of fibrosis but without honeycombing, defined as possible UIP pattern by the official ATS/ERS/JRS/ALAT statement [2]. The 7 patients with a CT pattern of possible UIP did not give their consent to lung biopsy, necessary to make a definitive diagnosis and we have indicated these patients as indeterminate IIP. Table 1 describes the patients' characteristics. All patients underwent pulmonary function tests at the time of diagnosis. The tests were performed according to current guidelines [21] using an Elite series plethysmograph (Medical Graphics, St Paul, Minnesota, USA).

**Table 1:** Demographic and functional characteristics of the study subjects.

	Pulmonary fibrosis (N = 19)	Controls (N = 11)
Number of subjects	19	10
Age, yrs (mean ± SD)	71±8	51±18*
Gender, M/F	12/7	6/5
Smoking habit, Yes/Ex/No	1/9/9	4/3/4
Pulmonary function tests		(only 6 patients)
FVC, L (mean ± SD)	2.59±0.57	3.47±2.03
FVC, % predicted (mean ± SD)	86.4±18.5	106.7±21.3*
FEV1, L (mean ± SD)	2.17±0.45	2.53±1.30
FEV1, % predicted (mean ± SD)	93.0±21.7	103.5±31.9
TLC, L (mean ± SD)	4.22±1.11	6.85±2.30*
TLC, % predicted (mean ± SD)	74.3±20.1	113.8±17.5*
DlCO, mL/min/mmHg (mean ± SD)	14.0±3.8	22.1±11.4*
DlCO, % predicted (mean ± SD)	61.2±14.4	79.2±16.3*

* p<.05.

No patients were taking steroids, NAC, pirfenidone. One patient was under warfarin and had stopped it 6 days prior to bronchoscopy per standard procedure.

### MP isolation from BALF

Bronchoscopy was performed in all patients with a fiberoptic bronchoscope under topical lidocaine; BAL was performed by instilling 50-mL of sterile saline solution in one pulmonary segment of the middle lobe or lingula. The liquid recovered was filtered and centrifuged for 5′ at 350 x g to remove cells and subsequently for 10′ at 1800 x g to eliminate big debris. The supernatant was stored at −80°C and subsequently used for MP analysis. In experiments designed to analyze MP-associated TF activity, an aliquot of the BALF (12 mL) was submitted to ultracentrifugation for 2 h at 100,000 x g, and the pellet was resuspended in 250 µL of sterile saline solution.

### Measurement of MP

MP were detected in the conditioned medium of A549 cells, human bronchial epithelial cells (HBEC) and in BALF (see below) using the Zymuphen MP-activity kit according to the manufacturer's instructions and expressed as phosphatidylserine (PS) equivalents. Briefly, the assay is based on the property of annexin-V, immobilized onto plastic wells, to bind PS. A549 or HBEC supernatant or BALF was added to the wells and, after extensive washing, captured MP were detected by the addition of FVa, FXa, Ca^2+^ and prothrombin. Under the conditions used, the rate of thrombin formation is limited by PS availability and is therefore proportional to MP concentration. A chromogenic substrate was finally added to quantify thrombin concentration with a microplate reader (Titertek Multiskan MCC ELISA reader; Flow Laboratories, McLean, VA). Known amounts of PS were used to obtain a standard curve [22].

### Assessment of MP-associated TF activity

TF activity was measured in MP derived from A549 and BALF by a one-stage clotting time assay as described [22]. Briefly, normal, MP-free, human plasma (100 µL) is added to MP (100 µL) in a 37°C bath. CaCl_2_ (100 µL; 25 mM) is then added and time to formation of a visible clot upon recalcification is recorded. The results are expressed in arbitrary units (AU) of procoagulant activity by comparison with a standard curve obtained using a human brain thromboplastin standard. An anti-human TF antibody was used to assess the specificity of the test [23].

### Cell culture

Cells of the human alveolar epithelial line, A549, (American Type Culture Collection, CCL-195), were kindly provided by Dr. R. Danesi, University of Pisa, Pisa, Italy. A549 cells were maintained in RPMI supplemented with 10% (vol/vol) FBS, 100 U/mL of penicillin, and 100 µg/mL of streptomycin in a humidified 95% air- 5% CO_2_ atmosphere at 37°C.

HBEC were obtained from subjects undergoing diagnostic bronchoscopy as previously described [24]. Briefly, after the patient's informed consent to the procedure was received, the fiber-optic bronchoscope was positioned at the level of the carina and/or the level of second- or third-order bronchial branchings. The use of local anesthetics was kept as low as possible to minimize their effects on cell viability. Four to six brushings of grossly normal bronchial mucosa were regularly obtained. The cells were then removed from the brush by vortexing in Ham's F-12 medium-10% FBS. The cells were brought to the laboratory in ice and incubated with DNase (50 g/ml) to eliminate clumping. After a wash with ice-cold, serum-free Ham's F-12 medium, the cellswere resuspended in BEGM and plated on Vitrogen 100-coated culture flasks. Indirect immunofluorescence with anti-cytokeratin antibodies confirmed the epithelial origin of the cells. Cells used in the specific experiments reported here were obtained from a patient with peripheral lung cancer undergoing diagnostic bronchoscopy and were harvested from the contralateral main bronchus. The cells were used at passages 3–4.

### Lung epithelial cells stimulation and MP isolation from lung epithelial cells conditioned medium

A549 and HBEC were incubated with H_2_O_2_ (100 µM) or control buffer in the presence or in the absence of NAC (1 mM), in serum free medium. Following a 20 hour incubation, the conditioned medium was collected, cleared by centrifugation at 14,000 x g for 5′ at room temperature to remove dead cells and big fragments. In experiments designed to investigate MP-associated TF activity, conditioned medium (5 mL) was further purified by ultracentrifugation (100,000 x g) and the pellet obtained was resuspended in 125 µL of sterile saline solution. A549 cells were recovered at 2, 4, and 20 hours for RNA extraction.

### Real Time PCR

The sense and antisense primers for human TF, RPL 11, RPL 13 and hypoxanthine phosphoribosyltransferase (HPRT) were obtained from Invitrogen (Milan, Italy) and had the following sequences: TF forward: TTGGCAAGGACTTAATTTATACAC; TF reverse: CTGTTCGGGAGGGAATCAC; RPL 11 forward: ACTTCGCATCCGCAAACTCT; RPL 11 reverse: TGTGAGCTGCTCCAACACCT; RPL 13 forward: CCTGGAGGAGAAGAGGAAAGAGA; RPL 13 reverse: TTGAGGACCTCTGTGTATTTGTCAA; HPRT forward: AGACTTTGCTTTCCTTGGTCAGG; HPRT reverse: GTCTGGCTTATATCCAACACT TCG.

Real-time PCR was performed using iQ SYBR Green Supermix on the MiniOpticon Two-Color Real-time PCR detection System (Bio-Rad, Hercules, CA, USA). PCRs were performed in duplicate and HPRT was coamplified to normalize the amount of RNA added to the reaction. All data were analyzed using the OpticonMonitor3® software (Bio-Rad, Hercules, CA, USA). To compare the expression of mRNA levels among different samples, the relative expression of mRNA levels was calculated using the comparative ΔCT (threshold cycle number) method [25]. Briefly, the following formula was used: 2-ΔΔCT, where ΔΔ*CT* is the difference in CT between the gene of interest and HPRT, RLP11, RLP13 and *CT* for the sample  =  *CT* for the actual sample - *CT* of the lowest expression sample. The amplification efficiencies of the primers pairs were determined running serial dilutions of the cDNA. Both target and reference genes were amplified with efficiencies near 100% with a r^2^ value of 0.99.

### Reagents and Kits

RPMI 1640 medium, penicillin, streptomycin, L-glutamine, trypsin/EDTA, Dulbecco Phosphate Buffer Saline (PBS), Fetal Bovine Serum (FBS), N-acetyl-cysteine (NAC), Ham's F12, DNAse and anti-cytokeratin peptide-18 antibodies were obtained from Sigma (Milan, Italy). BEGM Bullet Kit was obtained from Cambrex (Caravaggio, BG, Italy). Vitrogen was obtained from Tebubio (Milan, Italy). iScript cDNA synthesis Kit and iQ SYBR green supermix were obtained from Bio-Rad (Hercules, CA, USA). NucleoSpin RNA II was obtained from Machery-Nagel (Duren, Germany). The Zymuphen MP-activity kit was from Hyphen BioMed, Neuville-sur-Oise, France. All standard chemicals were obtained from the hospital pharmacy and were of the best grade available.

### Statistical analysis

Data analysis was performed with Prism software, version 5.0a (GraphPad, La Jolla, CA, USA). Comparisons among cell treatments were performed with independent measures ANOVA followed by Tukey's correction; comparisons between patient groups were performed with the Mann-Whitney test. P values below .05 were considered statistically significant.

## Results

### PF is associated with an increase in MP-associated TF activity

**Figure 1:**
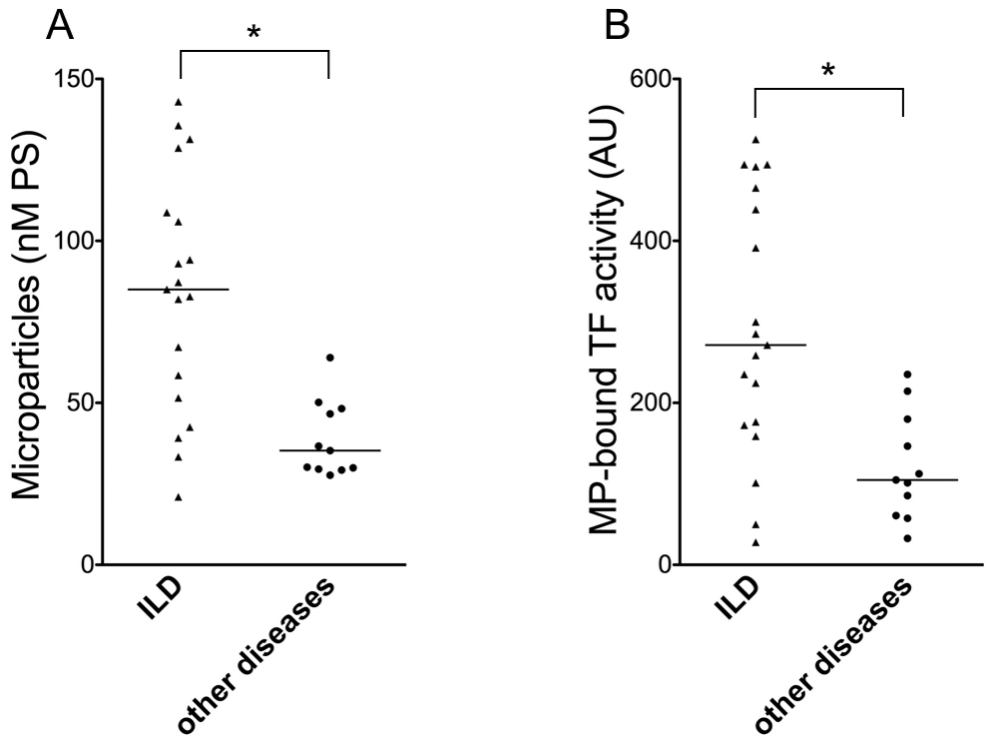
MP content and MP-associated TF activity in PF patients and controls. A) Total MP concentration, expressed as nM PS, in BALF of patients with PF and in control subjects with other pathologies. Bars represent median values; *p<0.05 (Mann-Whitney test) B) MP-associated TF activity of BALF of patients with PF and in control subjects with other pathologies. Bars represent median values; *p<0.05 (Mann-Whitney test)

**Figure 2:**
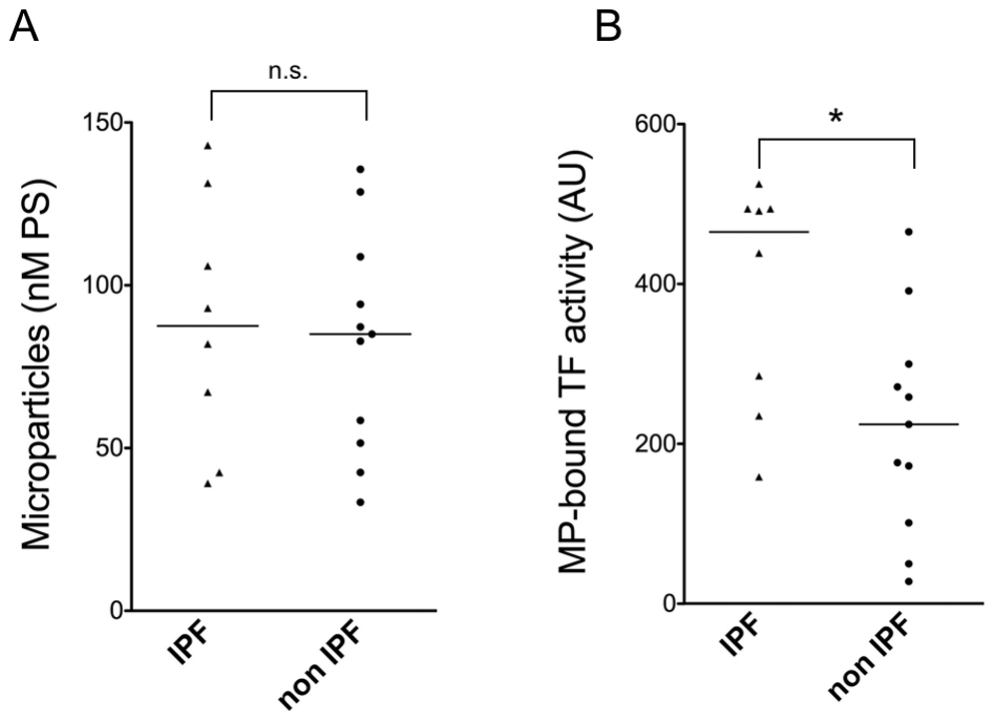
MP content and MP-associated TF activity in IPF and non IPF patients. A) Total MP concentration, expressed as nM PS, in BALF of patients with non-IPF and IPF CT scan patterns. Bars represent median values; n.s.: non significant (Mann-Whitney test) B) MP-associated TF activity of BALF of patients with non-IPF and IPF CT scan patterns. Bars represent median values; *p<0.05 (Mann-Whitney test).

Total MP were measured in PF and non-PF patients. As shown in figure 1, there is an increased number of MP in PF patients (1A). A comparison between the two groups of patients also showed a statistically significant increase in MP-associated TF activity in PF patients (1B). We also compared both total MP and MP-associated TF activity between two different groups of PF, IPF and non-IPF. Despite the small sample size, there is a statistically significant increase of MP-associated TF activity between the two groups of patients. No trend toward a difference was observed for total MP (fig. 2).

### MP-associated TF activity but not total MP correlates with the degree of functional impairment

**Figure 3:**
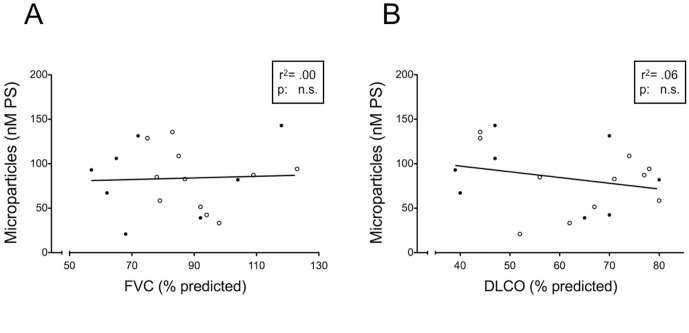
Correlation between MP content and lung function parameters in PF patients. A) Correlation between total MP and FVC (expressed as % predicted) in patients with PF (linear regression, r^2^ = 0.00, non significant). B) Correlation between total MP and DlCO (expressed as % of predicted) in patients with PF (linear regression, r^2^ = 0.06, non significant). Filled circles: IPF; open circles: non-IPF

**Figure 4:**
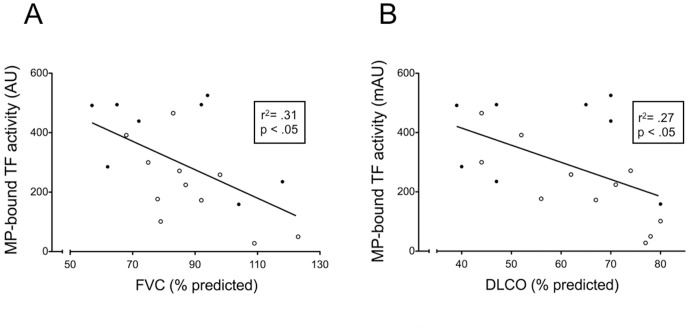
Correlation between MP-associated TF and lung function parameters in PF patients. A) Correlation between MP-associated TF activity and FVC (expressed as % predicted) in patients with PF (linear regression, r^2^ = 0.31, p = 0.0137). B) Correlation between MP-associated TF activity and DlCO (expressed as % of predicted) in patients with PF (linear regression, r^2^ = 0.27, p = 0.0217). Filled circles: IPF; open circles: non-IPF.

To further evaluate the potential role of MP and MP-associated TF activity in PF, we investigated their correlation with functional impairment. There was no correlation between total MP and either forced vital capacity (FVC) (% predicted) and diffusion capacity for CO (DlCO) (% predicted) (fig. 3). In contrast, the amount of MP-associated TF activity showed a statistically significant correlation with the reduction of both and DlCO (fig. 4).

### Oxidative stress induces the release of MP by lung epithelial cells

**Figure 5:**
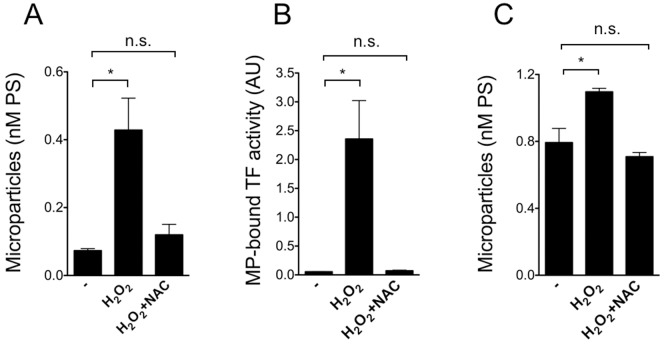
Total MP content and MP-bound TF activity in the conditioned media of lung epithelial cells exposed to H_2_O_2_. A) A549 cells were incubated in the absence or in the presence of H_2_O_2_ (100 µM) with or without preincubation (30 min) with NAC (1 mM) for 20 hours in serum free medium. Total MP were assessed in the consditioned medium based on PS concentration. Data are mean ± SEM from 6 consecutive, independent experiments; *p<0.05 (ANOVA followed by Tukey's correction). B) A549 cells were treated as described above. The conditioned medium was submitted to ultracentrifugation and the pellet resuspended and tested for TF activity with a one stage clotting assay. Data are mean ± SEM from 6 consecutive, independent experiments; *p<0.05 (ANOVA followed by Tukey's correction). C) NHBEC cells were incubated in the absence or in the presence of H_2_O_2_ (100 µM) with or without preincubation (30 min) with NAC (1 mM) for 20 hours in the medium. Total MP were assessed in the conditioned medium based on PS concentration. Data are mean ± SEM from 3 consecutive, independent experiments; *p<0.05 (ANOVA followed by Tukey's correction).

To begin to investigate the potential mechanisms of procoagulant MP generation in the context of PF, we used a stimulus classically associated with the disease, namely oxidative stress. Figure 5A shows that exposure of A549 cells to H_2_O_2_ caused a significant increase in MP. Preincubation with the antioxidant NAC reverted the effect. Incubation of A549 cells with NAC alone did not affect MP generation (not shown). We then investigated whether MP shed by A549 cells upon oxidation contain functionally active TF. As shown in Fig. 5B, H_2_O_2_ induces a marked, statistically significant increase in TF-mediated procoagulant activity associated with MP. A monoclonal antibody to TF inhibited most of the procoagulant activity confirming its identity with TF (not shown). Pretreatment of A549 with NAC significantly reduced the amount of MP-associated TF activity. Similar results were obtained with NHBEC in primary culture (fig. 5C).

### Oxidative stress does not modulate TF mRNA synthesis by A549 cells

**Figure 6:**
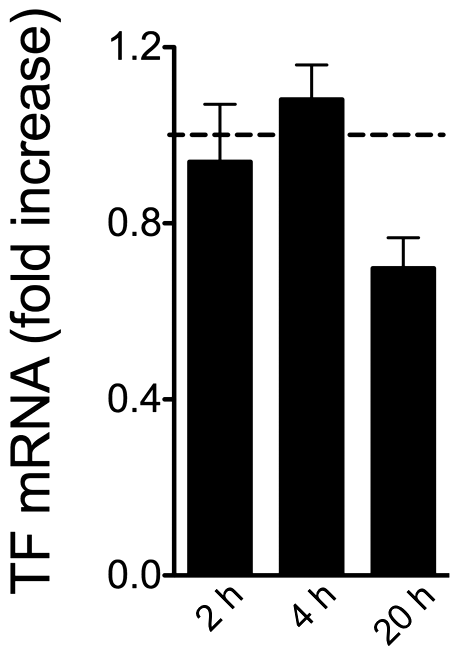
H_2_O_2_ does not modulate TF mRNA synthesis by A549 cells. A549 cells were treated as described before. Total mRNA was extracted at the indicated time points and TF mRNA analyzed by RT-PCR. Data are from 3 consecutive, independent experiments and represent mean ± SEM of fold increase over mRNA detected in unstimulated cells at the different time points.

Because an increase in MP-associated TF activity could either derive from an increased number of shed MP, each expressing similar amounts of TF, or from an increased synthesis of TF by the parental cell prior to MP generation, we investigated the role of H_2_O_2_ in modulating TF mRNA expression. As shown in fig. 6, incubation of A549 cells with H_2_O_2_ does not cause an increase in TF-mRNA.

## Discussion

Originally considered laboratory artifacts or, at most, cell debris devoid of physiological significance, MP are now recognized as significant participants in several physiological and pathophysiological conditions. A role for MP has been demonstrated, for example, in inflammation [26], including lung inflammation [11], [27]–[29] The role of MP in blood coagulation is also well characterized. Because negatively charged phospholipids are required for the assembly of the multimolecular complexes, termed tenase and prothrombinase, responsible for FX and prothrombin activation, MP, that express PS on their outer membrane, represent an ideal surface for these reactions. Furthermore, some MP express TF on their surface, which enhances their procoagulant potential [30]. Accordingly, MP are increased in a variety of diseases characterized by coagulation abnormalities [31]–[34].

Based on the above considerations, and on the emerging role of the activation of blood coagulation in PF, we investigated the potential role of procoagulant, TF-bearing MP in the pathogenesis of the disease.

We measured MP and MP-associated TF in BALF of patients with PF and of patients undergoing bronchoscopy for different diseases. Total MP (expressed as PS concentration) are significantly increased in PF patients; however, the difference between PF and non-PF patients is more striking when MP-associated TF activity, rather than total MP, is measured. When PF patients were divided into IPF and non-IPF, based on CT features characteristic of UIP [2], we observed no difference in total MP content; in contrast, MP-associated TF activity was significantly higher in IPF patients. Finally, the observation that MP-associated TF activity is related to functional impairment lends further support to a role of TF bearing MP in the development of PF.

MP generation is a regulated phenomenon, and exposure of a cell to different stimuli induces the shedding of MP with different membrane composition [35]. Our observation that total MP number is less related to the nature and severity of PF than MP-associated TF activity is consistent with the hypothesis that stimuli leading to a fibrotic response specifically induce the generation of TF-containing MP.

Oxidative stress has been recognized to contribute to PF progress. Indeed, high dose NAC appears to preserve vital capacity and DlCO in IPF patients [36]. Our observation that H_2_O_2_ induces the generation of procoagulant MP from lung epithelial cells is at least consistent with a potential role of these vesicles in the pathogenesis of PF. Our data do not rule out that other cell types (such as fibroblasts) might be a source of TF bearing MP.

PF is a complex disease whose pathogenesis is poorly understood. A role for the activation of blood coagulation and for PAR-1-mediated signaling by FXa is clearly emerging [37]; our data add to the recently proposed model that describes the contribution of locally synthesized FX to the fibrotic response in lung injury [20] demonstrating that MP-associated TF activity is increased in patients with PF and is related to the severity of the disease. Thus, the model can be expanded to predict that lung cells exposed to fibrogenic stimuli (e.g. oxidative stress) shed TF-bearing MP which can then bind to locally generated FX and FVIIa leading to FX activation. Pharmacological inhibition of MP shedding [38] might represent a future strategy for PF therapy.
